# Supplementary material for: Patterns of Intron Gain and Loss in Fungi
Source: PLoS Biol. 2004 Nov 30;2(12):e422. doi: 10.1371/journal.pbio.0020422 (PMC532390; doi:10.1371/journal.pbio.0020422)
Supplement: Table S1 — Also available at http://genes.mit.edu/NielsenEtAl/. (4.3 MB ZIP). [file pbio.0020422.st001.zip › NielsenEtAl/html/886.html]

AN0676.1.NCU03954.1.MG00961.1.FG09993.1


```
 CLUSTAL W (1.82) Multiple Sequence Alignments - Introns Inserted


Sequence 1: NCU03954.1	461 aa
Sequence 2: FG09993.1	493 aa
Sequence 3: MG00961.1	486 aa
Sequence 4: AN0676.1	454 aa
Alignment Length: 517 aa
Number Identitical Residues: 358 aa
Alignment Score (without introns) 15086


MG00961.1 	MPR2EIITIQAGQCGNSI1GSQFWQQLCQEHGINQDGNLEDFATEGGDRKDVFYYQ~SDD
NCU03954.1	MPR2EIITIQAGQCGNSI1GSQFWQQLCLEHGISQDGTIEDFATEGGDRKDVFFYQ~SDD
FG09993.1 	MPR2EIITIQAGQCGNSI1GSQFWQQLCQEHGISQDGNLEDFATEGGDRKDVFYYQ~SDD
AN0676.1  	MSR~EIITIQAGQCGNNV1GSQFWQQLCLEHGISQDGNLEEFATEGGDRKDVFFYQ0SDD
          	*.* ************.: ********* ****.***.:*:************:** ***

MG00961.1 	TRYIPRAILIDLEPR0VINGIQTGPYKNIYNPENFYVGKNGVGAANNWGDGYQTGESVHE
NCU03954.1	TRYIPRSILIDLEPR~VINTIQTGPYRNIYNPENFYVGKSGLGAGNNWGDGYQTGEQVHE
FG09993.1 	TRYIPRAILIDLEPR0VINGIQTGPYKNIYNPENFYVGKDGVGAANNWGDGYQSGEAVYE
AN0676.1  	TRYIPRAILLDLEPR0VLNGIQSGPYKNIYNPENFFIGQQGIGAGNNWGAGYAAGEVVQE
          	******:**:***** *:* **:***:********::*:.*:**.**** ** :** * *

MG00961.1 	DIMEMIDREADGSDSLE0GFMMLHSIAGGTGSGLGSFLLERLNDRFPKKIIQTYSVFPDT
NCU03954.1	EIMEMIEREADGSDSLE~GFMMLHSIAGGTGSGLGSFLLERLNDRFPKKIIQTYSVFPDT
FG09993.1 	DIMEMIDREADGSDSLE0GFMMLHSIAGGTGSGLGSFLLERLNDRFPKKIIQTYSVFPDT
AN0676.1  	EVFDMIDREADGSDSLE0GFMFLHSIAGGTGSGLGSFLLERMNDRFPKKLIQTYSVFPDT
          	::::**:********** ***:*******************:*******:**********

MG00961.1 	QNAGDVVVHPYNSILAMRRLTQNADSV~VVLDNGALSHIAADRLHVQEPSFQQTNQL0VS
NCU03954.1	TSAGDVVVHPYNSLLAMRRLTQNADSV~VVLDNGALSHIAADRLHVQEPSFQQTNQL~VS
FG09993.1 	TNAGDVVVHPYNSILSMRRLVQNADSV~VVLDNGALSHIAADRLHVQEPSFQQTNQL~VS
AN0676.1  	Q-AADVVVNPYNSLLAMRRLTQNADSV0VVLDNAALSRIVADRLHVQEPSFQQTNRL0VS
          	  *.****:****:*:****.****** *****.***:*.***************:* **

MG00961.1 	-------------------------------TVMSASTTTLRYPGYMHNDLVSILASLIP
NCU03954.1	-------------------------------TVMSASTTTLRYPGYMHNDLVSILASLIP
FG09993.1 	TTVLFNYQIHVMKAICGCVSQDERTNNCQVATVMSASTTTLRYPGYMHNDLVSILASLIP
AN0676.1  	-------------------------------TVMSASTTTLRYPGYMHNDLVGIIASLIP
          	                               *********************.*:*****

MG00961.1 	TPKCHFLMTSYTPFTGDQVEQAKTVRKTTVLDVMRRLLQPKNRMVSTIPGKKSCYISILN
NCU03954.1	TPRCHFLMTSYTPFTGDQVEQAKTVRKTTVLDVMRRLLQPKNRMVSTVPGKKSCYISILN
FG09993.1 	TPRCHFLMTAYTPFTGDQVEQAKTVRKTTVLDVMRRLLQPKNRMVSTVPGKKSCYISILN
AN0676.1  	TPRSHFLLTSYTPFTGDNIDQAKTVRKTTVLDVMRRLLQPKNRMVSINPSKSSCYISILN
          	**:.***:*:*******:::**************************  *.*.********

MG00961.1 	VIQGEVDPTDVHKSLLRIRERRLATFIPWGPASIQVALTKRSPYIPMSHRVSGLMLANHT
NCU03954.1	VIQGDVDPTDVHKSLLRIRERRLATFIPWGPASIQVALTKRSPYVTMAHRVSGLMLANHT
FG09993.1 	VIQGEVDPTDVHKSLLRIRERRLATFIPWGPASIQVALTKRSPYIPMSHRVSGLMLANHT
AN0676.1  	IIQGEADPTDVHKSLLRIRERRLASFIPWGPASIQVALTKKSPYIQNTHRVSGLMLANHT
          	:***:.******************:***************:***:  :************

MG00961.1 	SIAT~VRNTFVVFLITEFILTKCALNVLQLFKRIVRQYDGMRKRNAFIEGYKKTQPFSEN
NCU03954.1	SIAT0------------------------LFKRIVRQYDGMRKRNAFMEAYKKTAPFSEN
FG09993.1 	SIAT0------------------------LFKRILRQYDGMRKRNAFMEGYKKTAPFSEN
AN0676.1  	SVAT~------------------------LFKRIVQQYDRLRKRNAFLEQYKKEAPFQDG
          	*:**                         *****::*** :******:* ***  **.:.

MG00961.1 	LDEFDEARQVVSDLIAEYEAAEDANYLNPDAGEPGTSAETDRRMG
NCU03954.1	LNEFDEAREVVMDLIADYEAAEDANYLNPELGEN-ASADTDKRMA
FG09993.1 	LNEFDEARQVVADLIGEYEAAEDADYLNPDAGEKPTSIETDRRVG
AN0676.1  	LDEFDEARAVVMDLVGEYEAAERENYLDPDAGKDEVGV-------
          	*:****** ** **:.:*****  :**:*: *:  ..
```
